# Emergent Dirac Fermions in Epitaxial Planar Silicene Heterostructure

**DOI:** 10.1021/acs.nanolett.3c04046

**Published:** 2024-01-05

**Authors:** Marek Kopciuszyński, Agnieszka Stȩpniak-Dybala, Ryszard Zdyb, Mariusz Krawiec

**Affiliations:** Institute of Physics, M. Curie-Sklodowska University, Pl. M. Curie-Skłodowskiej 1, 20-031 Lublin, Poland

**Keywords:** 2D materials, silicene, Dirac fermions, ARPES, STM, DFT

## Abstract

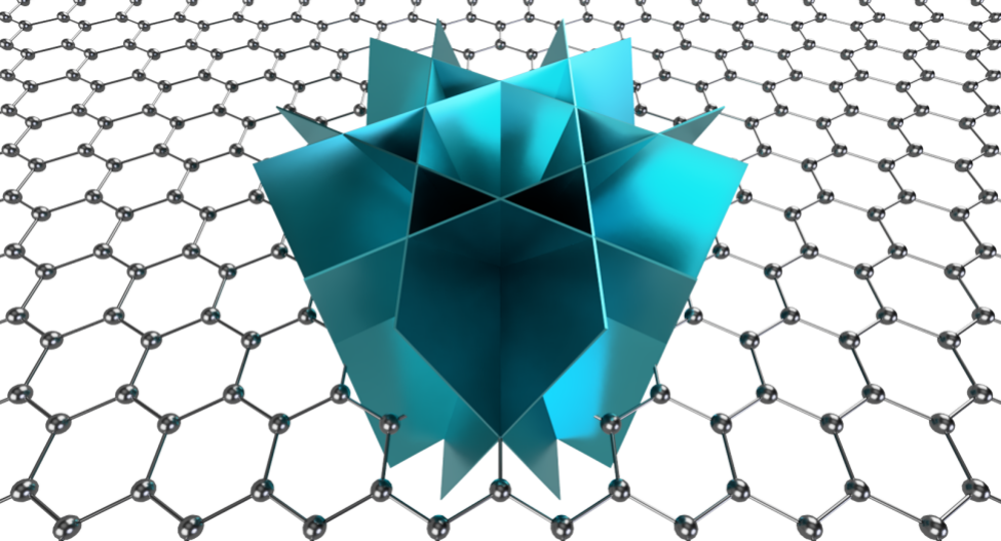

Silicene, a single layer of Si atoms, shares many remarkable electronic properties with graphene. So far, silicene has been synthesized in its epitaxial form on a few surfaces of solids. Thus, the problem of silicene–substrate interaction appears, which usually depresses the original electronic behavior but may trigger properties superior to those of bare components. We report the direct observation of robust Dirac-dispersed bands in epitaxial silicene grown on Au(111) films deposited on Si(111). By performing in-depth angle-resolved photoemission spectroscopy measurements, we reveal three pairs of one-dimensional bands with linear dispersion running in three different directions of an otherwise two-dimensional system. By combining these results with first-principles calculations, we explore the nature of these bands and point to strong interaction between subsystems forming a complex Si–Au heterostructure. These findings emphasize the essential role of interfacial coupling and open a unique materials platform for exploring exotic quantum phenomena and applications in future-generation nanoelectronics.

Since the discovery of graphene with its extraordinary physical properties,^1,2^ much effort has been dedicated to searching for other two-dimensional (2D) materials.^3−6^ This opened a new era of condensed matter physics, revealing a plethora of intriguing quantum phenomena that arise when systems become thinner and thinner down to single-atom layers. Among them, the remarkable electronic properties, characterized by energy dispersions that deviate from the common free-electron-like quadratic dependence, opened a route to a rich realm of novel physics and technological innovations. The charge carriers in graphene behave like massless relativistic fermions showing a linear dispersion around the Fermi energy (*E*_F_) forming so-called Dirac cones at specific *k*-points in the reciprocal lattice.^1^ The field of Dirac cones has rapidly expanded into various classes of materials.^7^ One such class of 2D materials beyond graphene includes group 14 Xenes: silicene (Si), germanene (Ge), stanene (Sn), and plumbene (Pb).^6,8−10^

Silicene, a single layer of Si atoms arranged in a honeycomb lattice, has attracted a great deal of attention in recent years owing to its predicted outstanding electronic properties and the promise for the next-generation nanoelectronics.^6,11−13^ Although its free-standing equilibrium geometry is low buckled due to a mixed sp^2^ and sp^3^ hybridization state, the extraordinary electronic properties, characteristic of graphene, should also be preserved.^14,15^

Despite the theoretical predictions, the synthesis of silicene remains a great challenge. Experimentally, silicene for the first time has been successfully grown on a Ag(111) substrate.^16−19^ Since then, several attempts to fabricate silicene have been performed by using other templates, like ZrB_2_(0001), Ir(111), ZrC(111), Ru(0001), and Pb(111).^10,20−25^ All of these studies report the synthesis of silicene in the form of a lattice on a substrate, often reconstructed, although a quasi-free-standing epitaxial silicene and graphene-like form, i.e., the planar silicene, has also been synthesized.^26−29^ Other 2D allotropes of Si, including dumbbell silicene and Kagome lattice, have also been discovered.^30,31^

Usually, substrate interaction depresses the superior electronic properties of the isolated form. Indeed, research has failed to observe solid evidence of Dirac cones in epitaxial silicene grown on metallic substrates. The enhanced band hybridization between both subsytems is responsible for the absence of original Dirac cones, although the self-protection mechanism can operate.^32^ On the contrary, the presence of a substrate can be regarded as a method of functionalization^10^ and substantially alter the properties of the system or even induce new exotic phenomena. Thus, in some cases, epitaxial silicene can become a kind of emergent system. Emergence means the appearance of qualitatively new properties at the next level of the complexity. Here it concerns the properties or behavior of the system inaccessible to separate subsystems, i.e., silicene itself and the substrate itself. For example, six pairs of Dirac cones have been observed in silicene on Ag(111).^33^ They were located along the 1 × 1 surface Brillouin zone (BZ) boundary of Ag(111), suggesting that particles in these bands experience a strong Ag-derived electrostatic potential, which points to substantial interfacial effects. Indeed, the density functional theory (DFT) calculations have reproduced the experimentally observed 12 Dirac cones in this hybrid silicene/Ag(111) system and evidenced their mixed Si p_*z*_ and Ag sp character.^34,35^ Clearly, this unexpected behavior originates from the strong interaction between two system components combined into a single heterostructure.

In the context of emergent phenomena, planar silicene prepared on Au(111) films deposited on Si(111) appears as a prime candidate owing to its peculiar atomic structure.^27,28^ In particular, the planar layer is a part of a more complex Si–Au heterostructure, which additionally contains the rotated by 22° buckled silicene [or Si(111) layer] and a Au(-Si) layer as a spacer. Undoubtedly, such complexity of atomic structure should be reflected in the unusual behavior of charge carriers.

In this work, we report direct observation of robust Dirac-dispersed bands in epitaxial silicene grown on Au(111) films deposited on Si(111). By performing in-depth angle-resolved photoemission spectroscopy (ARPES) measurements, we reveal three pairs of one-dimensional (1D) bands with linear dispersion running in three different directions. In other words, we deal with 1D electron band structure (BS) in a 2D system. Such behavior demonstrates clearly that this Si–Au structure can be considered an emergent system. By combining these results with first-principles DFT calculations, we explore the nature of these bands and point to the importance of the interfacial effects and strong interactions between subsystems forming this complex Si–Au heterostructure.

Epitaxial growth of Au films on the Si(111) template results in different surface Si–Au structures, which can be controlled by the substrate temperature during or after deposition.^36^ The planar form of silicene can be attained at 560 K. The large-area scanning tunneling microscopy (STM) image of this structure is rendered in Figure 1a. Characteristic features, domain-like boundaries of deformed hexagonal shapes, are clearly visible, similar to previously observed structures.^27^ The STM apparent height of the hexagon boundaries yields ∼30 pm (Figure 1c) and confirms the continuous character of the planar silicene. Atomically resolved STM scans reveal the 3-fold symmetry of the surface structure, as marked in Figure 1b. The flat character of the surface is demonstrated in the line profile shown in Figure 1d. All of these findings match well literature reports of epitaxial planar silicene.^27^

**Figure 1:**
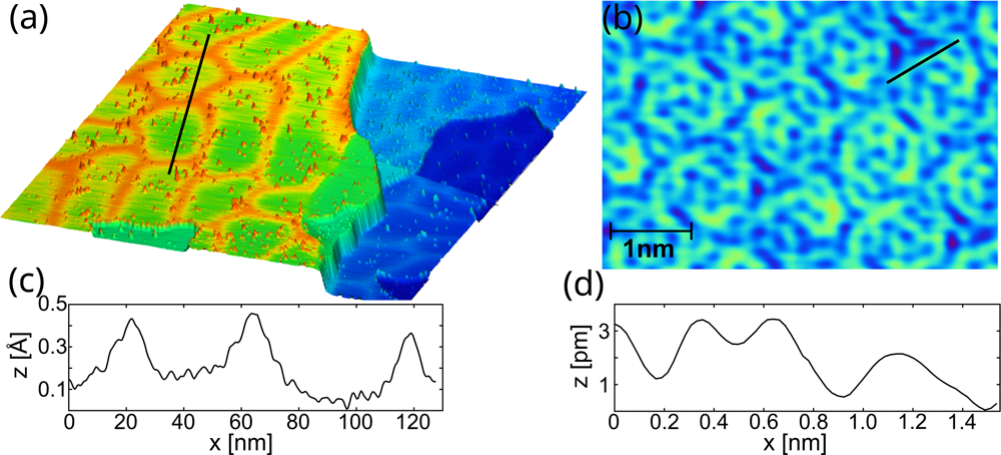
(a) Three-dimensional rendering of an STM topography image of planar silicene on Au(111) (*V*_bias_ = −2.0 V, and *I*_tunn_ = 150 pA). (b) Atomically resolved STM image (*V*_bias_ = 1.5 V, and *I*_tunn_ = 300 pA). (c) Line profile of the topography in panel a passing through hexagon boundaries. (d) Profile along the line marked in panel b, demonstrating the flat character of the top Si layer.

The homogeneous, macroscopically ordered surface opens up the possibility for an unambiguous ARPES study of the electronic structure. Panels a and b of Figure 2 show the band structure of epitaxial planar silicene on Au(111) measured along different momentum directions marked in panel c. A very bright steep band around the K point of the surface BZ is the bulk Au sp band. Furthermore, there are also V-shaped bands assigned as quantum well states (QWSs). The number of QWSs as well as their energy positions depend on the thickness of the Au slab. Both types of bands have already been evidenced previously.^37^ In addition, two bands with Dirac-like linear dispersion around the BZ center (Γ point) and crossing of the *E*_F_ at *k* = ±0.1 Å^–1^ are clearly visible. One can notice that Dirac cones meet at the Dirac point (DP) located at *E* = −0.75 eV, which suggests significant electron doping of the system. The group velocity is estimated to be approximately (1.34 ± 0.05) × 10^6^ m/s, which is more than twice that of quasi-free-standing epitaxial silicene on Ag(111).^26^ Note, however, that this value should not be exactly related to free-standing silicene, as these are completely different electronic bands.

**Figure 2:**
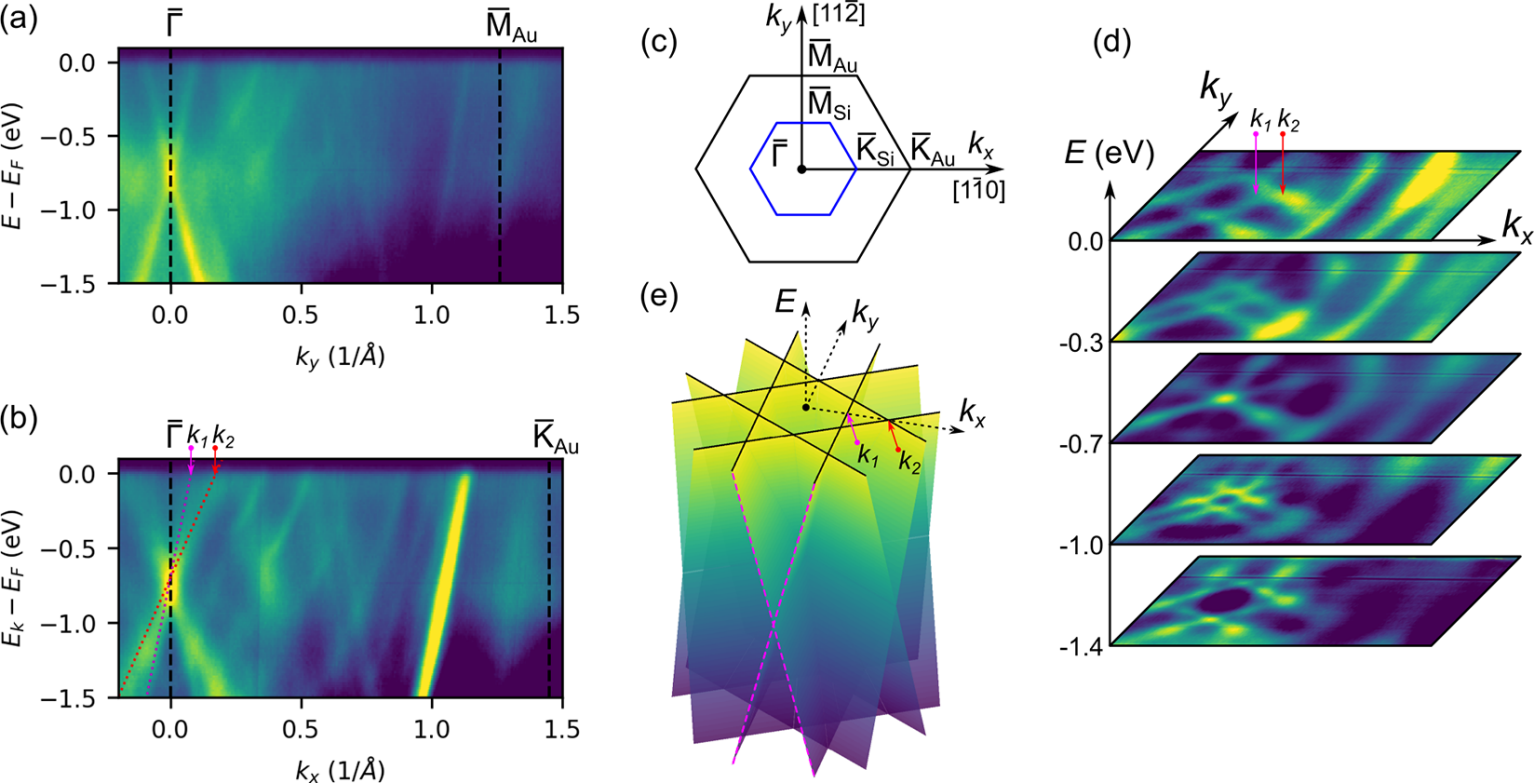
(a and b) Electronic band dispersions obtained by ARPES along the Γ–M and Γ–K directions of the surface Brillouin zone sketched in panel c. (d) Constant energy cuts showing the existence of three pairs of 1D Dirac bands and their evolution with energy. Data are presented as intensity maps, where the yellow color indicates higher values. (e) Schematic three-dimensional view of the 1D linear bands revealed in ARPES measurements.

An extensive ARPES data set in the form of *E*(*k*_*x*_, *k*_*y*_) is shown as constant energy cuts in Figure 2d. The Fermi surface (FS) exhibits three pairs of parallel lines running in three different Γ–M directions of the surface Brillouin zone. They intersect each other and form a Γ-centered hexagonal FS topology. The QWS states and bulk Au sp band, with the expected circular shape of the FS, are visible, too. With decreasing energy, this hexagonal shape reduces to a spot at the DP, and pairs of parallel lines to single lines. With a further decrease in energy, this spot grows into a hexagon again, and pairs of parallel lines are recovered, too. This behavior of linear 1D bands is schematically depicted in Figure 2e. More detailed evolution of the linear bands with energy is clarified in Figure S1. Note that from the energy cuts (also from the schematic three-dimensional BS) it is apparent that the BS along the Γ–K direction should exhibit two linear branches with different dispersions. One comes from the linear 1D band crossing the Γ–K line at point *k*_1_ (for this band, the group velocity was determined), and the other from 1D bands running in two other directions, intersecting exactly along the Γ–K line at point *k*_2_ (Figure 2d). Indeed, these branches are also visible in Figure 2b, although faintly.

The drastic dispersion differences between the 1D bands and other QWS or bulk bands clearly indicate their different origins. Additional arguments supporting this view can be deduced from temperature- and Au thickness-dependent ARPES experiments. The Dirac-like linear bands appear after the sample is annealed at 560 K, i.e., when the planar silicene structure has formed. Figure S2 shows the temperature evolution of the measured band structure. Furthermore, the shape and the position of these linear bands do not depend on the thickness of the Au slab, in strict contradiction to the QWS, as demonstrated in Figure S3. This undoubtedly proves the origin of these 1D Dirac bands other than the quantum size effect.

To understand our experimental results, we carried out first-principles DFT calculations of the epitaxial planar silicene. Following ref (28), we consider only the top part of the system consisted of the planar silicene, Si–Au layer, and the low-buckled silicene [or Si(111) plane]. The diffraction and microscopy experiments^28,36^ show that planar and low-buckled silicene layers are twisted by 22° with respect to each other and exhibit different lattice constants. All of this results in observed different superstructures: √3 × √3, √7 × √7, and √21 × √21. Au atoms separate planar and buckled silicene and stabilize the whole structure. The indirect proof for the formation of such layer may also come from chemically sensitive experiments.^38,39^ In calculations, we disregard the Au(111) slab and the Si(111) substrate, which will basically result in a shift of the energy bands, as discussed in ref (37). To make the discussion more clear, we further simplify our original model by limiting it to the √3 × √3 surface reconstruction, which is an approximate building block of a more complex real structure.^28^

Figure 3a shows the structural model of the √3 × √3 epitaxial planar silicene. After structural relaxation, the top Si layer retains its flat hexagonal honeycomb structure rather strongly interacting with the underneath Si–Au layer, as indicated by the small (2.21 Å) interlayer distance, certainly characteristic of true chemical bonds. Moreover, the distance between the Si–Au middle layer and the bottom silicene [or Si(111) layer], which yields a value of 2.80 Å, is also shorter than, for example, the corresponding separation between (111) planes of a bulk Si crystal (3.13 Å). This suggests that all three layers of the model cannot be separated into individual parts and should be considered as a whole. In other words, the epitaxial planar silicene must be investigated together with underneath layers, shown in Figure 3a.

**Figure 3:**
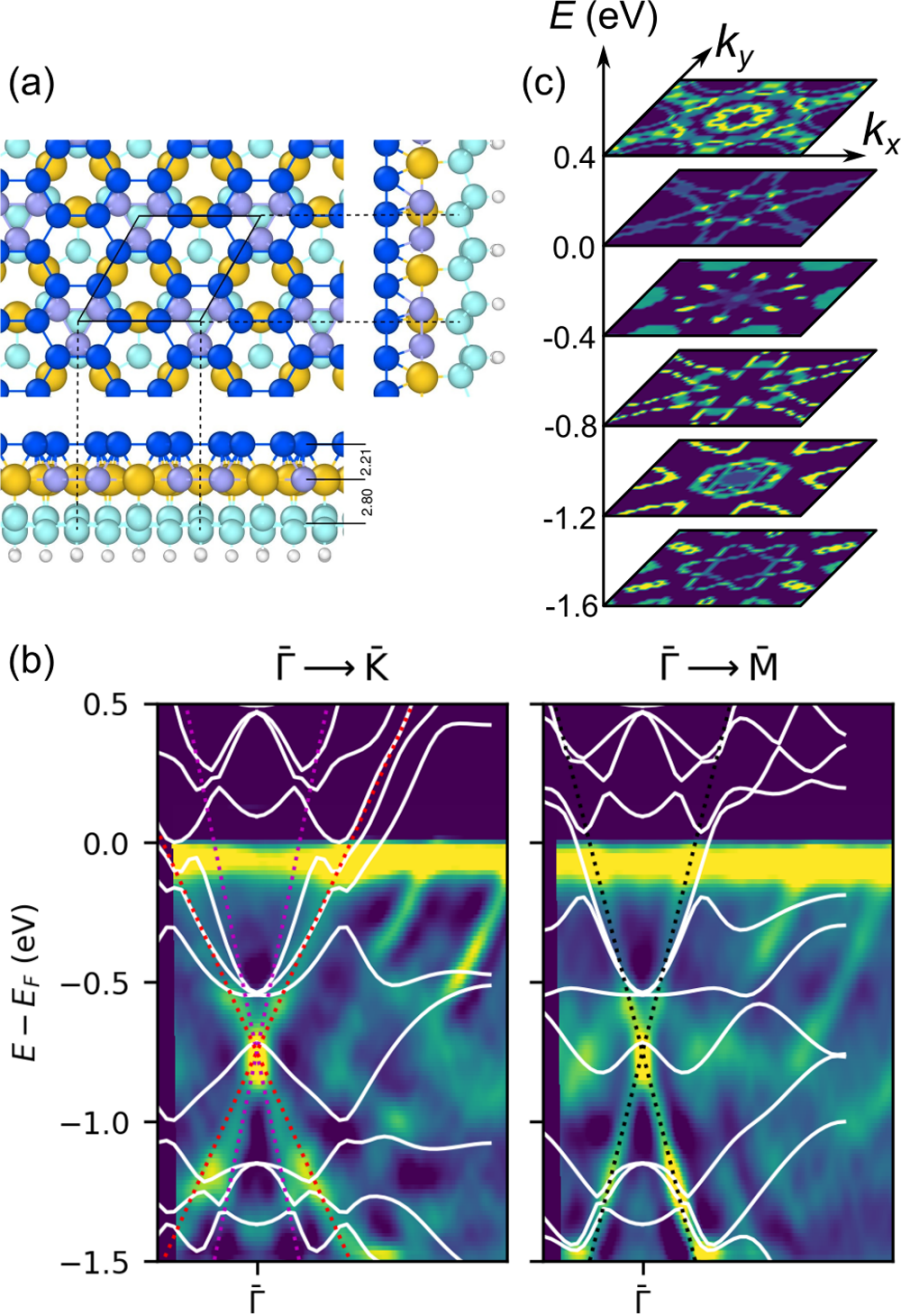
(a) Structural model of the Si–Au heterostructure consisting of (i) planar silicene (top), (ii) a Si–Au layer (middle), and (iii) low-buckled silicene (bottom layer). Below the bottom Si layer, saturating H atoms are present. Au atoms are colored orange, Si atoms shades of blue, and H atoms white. (b) Calculated electronic bands (white lines) superimposed on the ARPES spectra. Black dotted lines mark experimentally found 1D linear bands. Note two branches of linear dispersion along the Γ–K direction. (c) Theoretical constant energy cuts confirming the existence of three pairs of 1D Dirac bands.

The electronic band structure calculated for this model reproduces the main features of the ARPES spectra well, as shown in Figure 3b. In particular, linear bands with experimentally determined group velocity appear, and even two branches of these bands are resolved in the Γ–K direction. The DFT constant energy cuts, shown in Figure 3c, exhibit experimentally found hexagonal plus linear patterns.

To understand the origin of the 1D linear bands, we performed orbital analysis of the BS by projecting electronic bands onto orbitals with different symmetries of atoms belonging to three different layers of the structural model (Figure 3a). The results of the calculations are shown in Figure 4. It is evident that the orbital character of the 1D bands is complex, as different orbitals of different groups of atoms give rise to different parts of these bands. The main contribution to the upper branch comes from the s orbitals of the bottom layer with an admixture of p_*xy*_ orbitals of all three layers, p_*z*_ orbitals of the bottom Si layer, and the 5d states of Au in the middle layer. On the contrary, the lower branch has mainly p_*xy*_ character, but also s, with contributions from all three layers, but also p_*z*_ from the top Si layer, s from top and middle layers, and d from the middle layer. Such behavior confirms arguments of strong iterlayer interactions deduced from the geometry consideration.

**Figure 4:**
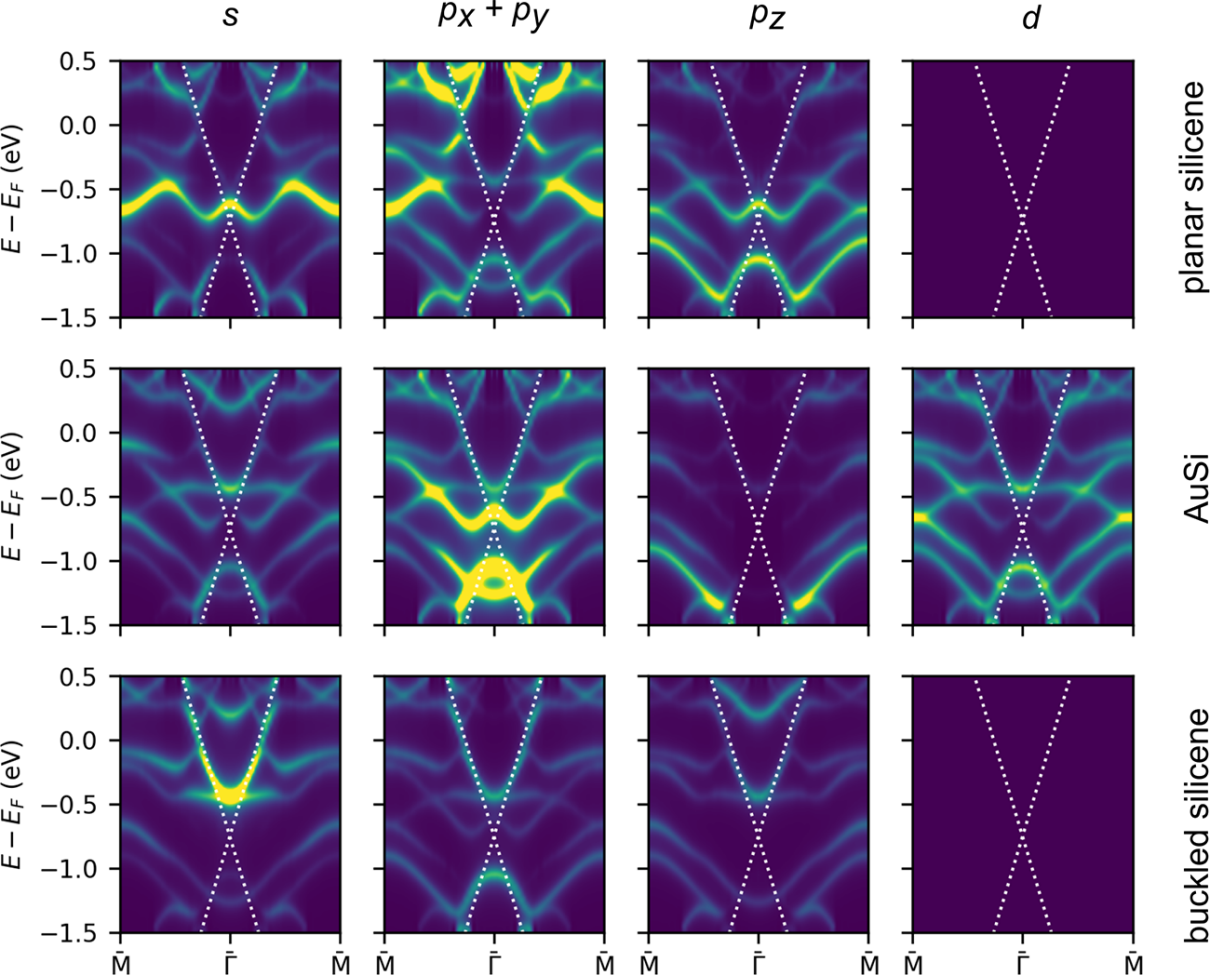
Calculated band structure shown as a relative contribution of s, p_*xy*_, p_*z*_, and d orbitals projected on the top (planar silicene), middle (AuSi), and bottom (buckled silicene) layer of the atomic structure shown in Figure 3a. Data are presented as intensity maps, where yellow color indicates higher values.

This shows the complexity of the BS, which results from strong interlayer interaction and hybridization of electronic states of all three layers of the structure, which, in turn, is a consequence of its hybrid nature and peculiar arrangement of the atomic layers. In particular, Au atoms in the Si–Au layer (Figure 3a) can be viewed as interpenetrating chains running in three high-symmetry surface directions producing 1D potential channels for electrons, while strong hybridization of the Au 5d states with the Si 3p_*xy*_ states makes electrons in all three layers prone to feeling this anisotropic Au-related potential. Thus, it is clear that the 1D bands cannot be assigned to the planar silicene or to any other building block of the Si–Au trilayer structure. They appear only in the full heterostructure, thus making it a kind of emergent system.

In summary, we have provided direct evidence for robust Dirac-dispersed 1D bands in epitaxial planar silicene grown on the Au(111) film deposited on the Si(111) substrate. We have revealed three pairs of such bands running in three different directions of an otherwise 2D system. We have demonstrated that these exotic band structures come into existence only when planar silicene is combined into a more complex three-layer heterostructure and are a consequence of strong interlayer interaction and hybridization of electronic states. These findings clearly indicate that the epitaxial planar silicene on Au films can be regarded as an emergent system and open a unique route for fundamental research and applications based on 2D Si systems.

## Methods

### Experimental Section

All STM and ARPES experiments were performed in two separate ultra-high-vacuum (UHV) chambers, both equipped with effusion cells, quartz microbalances, and the RHEED technique.

Samples were prepared according to the preparation procedure described in ref (27). In particular, an n-type Si(111) single crystal with a resistivity of 1 mΩ cm was used as the substrate. First, the Si(111) crystal was cleaned resistively to obtain the 7 × 7 reconstruction. Then it was passivated by Au or Ag, to afford 6 × 6 or √3 × √3 structures. Both reconstructions were used previously to improve the quality of ultrathin gold layers.^37,40^ The 6 × 6 Au reconstruction was prepared by deposition of 1.5 monolayer (ML) of Au, and the √3 × √3 Ag reconstruction was formed by deposition of 1 ML of Ag onto a clean Si(111) surface. One monolayer of Au or Ag refers to the density of atoms in a half of the bulk terminated Si(111) bilayer (7.84 × 10^14^ atoms/cm^2^). Gold was deposited on a Ag- or Au-passivated Si(111) surface at room temperature in an amount corresponding to several Au(111) monolayers. Afterward, the sample was gradually annealed up to 560 K using an electron beam heating technique or by direct heating. In both cases, the required structures have been obtained.

The STM measurements were performed at 77 K using the ScientaOmicron LT STM instrument with electrochemically etched W tips, and the STM images were processed using WSxM.^41^ The ARPES measurements were taken at 95 K using an unpolarized HeI line (21.2 eV), a Specs Phoibos 150 hemispherical analyzer equipped with a 2D multichannel plate detector, and a five-axis manipulator. The data were analyzed using a pyARPES library.^42^

### Calculations

First-principles DFT calculations were performed within revised Perdew–Burke–Ernzerhof for solids (PBEsol)^43^ generalized gradient approximation to the exchange-correlation interaction, as implemented in VASP (Vienna ab initio simulation package).^44,45^ The core electrons were treated within the projector-augmented wave method.^46^ The plane-wave energy cutoff for all calculations was set to 350 eV. The convergence criterion of the total energy for self-consistent field calculations was chosen to be 10^–7^ eV. The Brillouin zone was sampled by a 8 × 8 × 1 Monkhorst–Pack k-point grid including the Γ point.^47^

The silicene/Au(111) structure has been modeled by the planar and buckled silicene layers separated by a single-layer Si–Au layer. The bottom silicene [or Si(111) plane] was saturated with hydrogen atoms to mimic the rest of the substrate. The atomic positions were relaxed by a conjugate gradient method, until the largest force in any direction was <0.001 eV/Å. The lattice constant of the bottom Si layer was fixed at the bulk Si value.

## Supporting Information Available

ARPES measurements, including constant energy cuts, temperature evolution of the electronic bands, and the planar silicene structure synthesized on Au slabs of various thicknesses (PDF)
