# Supplementary material for: Emergent Dirac Fermions in Epitaxial Planar Silicene Heterostructure
Source: Nano Lett. 2024 Jan 5;24(7):2175–80. doi: 10.1021/acs.nanolett.3c04046 (PMC10885205; doi:10.1021/acs.nanolett.3c04046)
Supplement: Supplementary file 1 — nl3c04046_si_001.pdf [file nl3c04046_si_001.pdf]

Supporting Information

Emergent Dirac fermions in epitaxial planar silicene heterostructure

Marek Kopciuszyński, Agnieszka Stępniak-Dybala, Ryszard Zdyb, and Mariusz Krawiec*

Institute of Physics, M. Curie-Sklodowska University, Pl. M. Curie-Skłodowskiej 1, 20-031 Lublin, Poland

E-mail: mariusz.krawiec@mail.umcs.pl

Phone: +48 81 537 6146

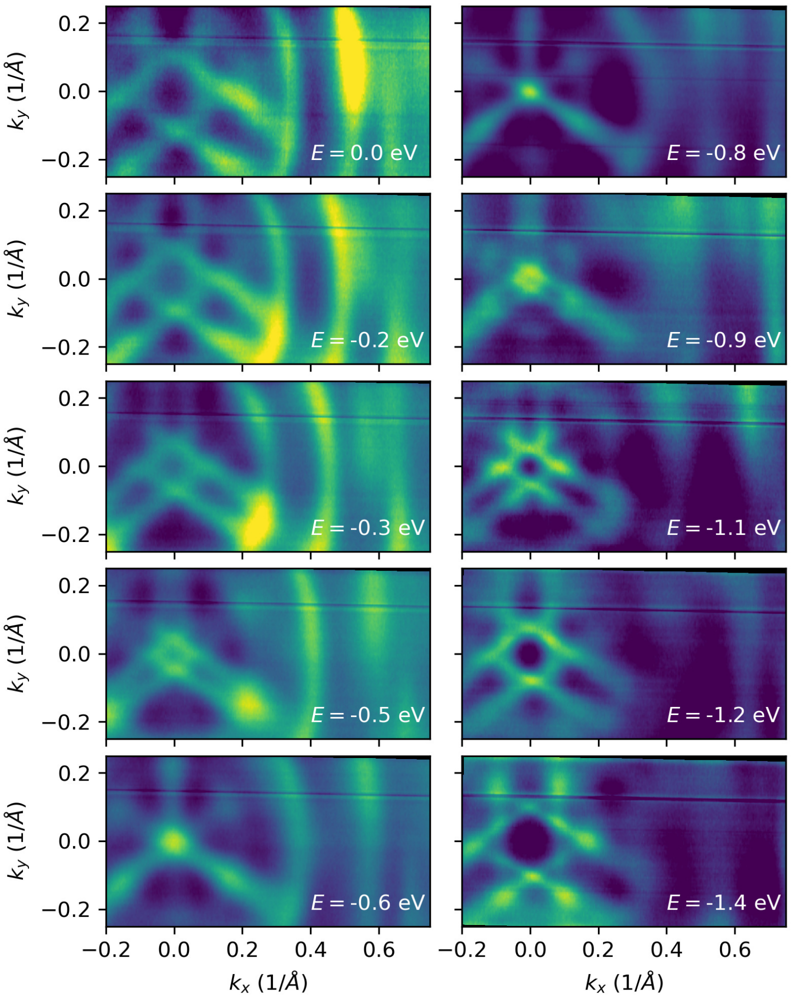

Figure S1: Constant energy cuts showing the existence of three pairs of 1D Dirac bands and their evolution with energy. Data are presented as intensity maps with yellow color indicating higher values.

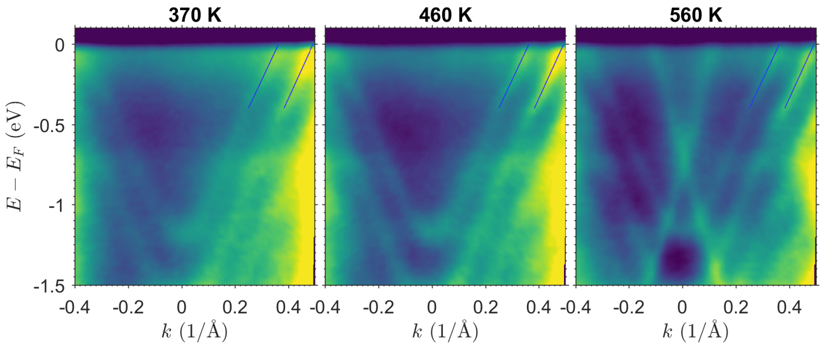

Figure S2: Temperature evolution of the electronic bands obtained by ARPES. Note that Dirac-like bands around the Γ point appear at temperature $T = 560$ K, while quantum well stats remain intact at all temperatures.

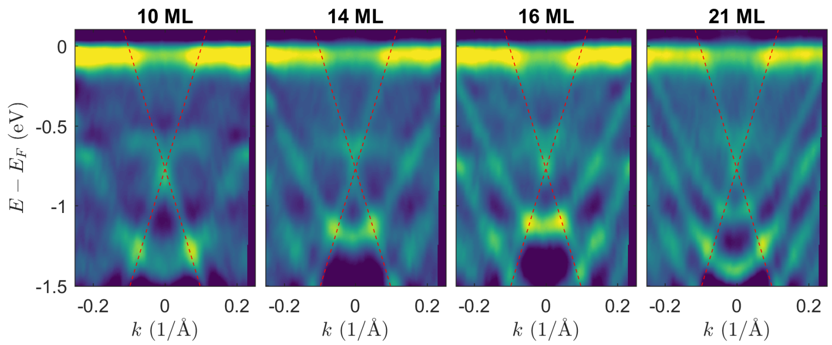

Figure S3: ARPES spectra collected for planar silicene structure synthesized on Au slabs of various thicknesses. Note that the Dirac-like band is insensitive to the Au thickness. On the other hand, positions of quantum well states, as expected, strongly depend on it.
